# Supplementary material for: The genome sequence of a gelechiid moth, Chionodes viduella (Fabricius, 1794) (Lepidoptera: Gelechiidae)
Source: Wellcome Open Res. 2026 Sep 14;11:641. [Version 1] doi: 10.12688/wellcomeopenres.27795.1 (PMC13650329; doi:10.12688/wellcomeopenres.27795.1)
Supplement: Peer review history

# Reviewer report: Reviewer response for version 1

**DOI:** 10.21956/wellcomeopenres.30588.r170183

**Published:** 2026-10-08

**Authors:** Jurate De Prins

**Affiliations:** 1 Science Committee, Global Biodiversity Information Facilities, Brussels, Belgium

This species does not occur in Belgium, so I am very grateful to the authors for providing me with diagnostic details in the subchapter 'Background'. On how they identified this species. Also, the photo this time shows the dorsal side of the insect and not a tiny moth shown ventral side up, as was the case with the *Caloptilia hemidactylella* sample for this journal. My plea remains that the photo in Fig. 1 is part of the research methodology, providing visual evidence of which species was studied for whole-genome sequencing. The first correct identification is crucial for further use of the whole-genome library.

 Also, a good point that the authors provided distribution and ecological details.

 The other point to which I kindly would like to pay attention is to determine if possible whether the sex determination mechanism is ZO or ZW.

 The third and last point is that the number of chromosomal pseudomolecules is reduced to 29 from the expected model n=30. Maybe there is a genetic explanation for that? Maybe one sex chromosome fused with an autosome?  If there is a genetic background for the reduced number, probably it would be interesting to mention.

Are the datasets clearly presented in a usable and accessible format, and the assembly and annotation available in an appropriate subject-specific repository?

Yes

Are sufficient details of the sequencing and extraction, software used, and materials provided to allow replication by others?

Yes

Are the rationale for sequencing the genome and the species significance clearly described?

Yes

Are the protocols appropriate and is the work technically sound?

Yes

Reviewer Expertise:

Taxonomy, Microlepidoptera, Bioinformatics, Karyology

I confirm that I have read this submission and believe that I have an appropriate level of expertise to confirm that it is of an acceptable scientific standard.

# Reviewer report: Reviewer response for version 1

**Published:** 2026-09-14

**Authors:** Automated Benchmarking

**Affiliations:** 1 Wellcome Sanger Institute, Hinxton, UK

As this article is a Genome Note, Automated Benchmarking has been used to determine whether the reported assembly passes three defined checks established by the Earth BioGenome Project: the raw and assembled data are publicly available, the Earth BioGenome Project three-part metric is met, and the BUSCO score passes the stated threshold. For Chionodes viduella (ilChiVidu1), all three checks passed. Data available: pass. ENA study PRJEB92262, raw-read accessions ERR15282566 and ERR15286550, and assembly accessions GCA_971853565.1 and GCA_971861385.1 are public. Earth BioGenome Project three-part metric: pass. Haplotype 1 is reported as 7.C.Q67, meeting the 6.C.Q40 reference standard. BUSCO: pass. Haplotype 1, which is proposed as the reference assembly for this species, has 99.7% complete BUSCOs (98.6% single-copy and 1.0% duplicated). BUSCO software version: 6.0.0; lineage dataset: lepidoptera_odb10. It meets the target of greater than 90% complete and single-copy BUSCOs. This decision uses values reported in the Genome Note and public accession records.

Reviewer Expertise:

NA

I confirm that I have read this submission and believe that I have an appropriate level of expertise to confirm that it is of an acceptable scientific standard.
